# Antidepressive Mechanisms of Probiotics and Their Therapeutic Potential

**DOI:** 10.3389/fnins.2019.01361

**Published:** 2020-01-14

**Authors:** Shin Jie Yong, Tommy Tong, Jactty Chew, Wei Ling Lim

**Affiliations:** Department of Biological Sciences, School of Science and Technology, Sunway University, Bandar Sunway, Malaysia

**Keywords:** microbiota-gut-brain axis, gut microbiota, major depressive disorder, probiotics, inflammation

## Abstract

The accumulating knowledge of the host-microbiota interplay gives rise to the microbiota-gut-brain (MGB) axis. The MGB axis depicts the interkingdom communication between the gut microbiota and the brain. This communication process involves the endocrine, immune and neurotransmitters systems. Dysfunction of these systems, along with the presence of gut dysbiosis, have been detected among clinically depressed patients. This implicates the involvement of a maladaptive MGB axis in the pathophysiology of depression. Depression refers to symptoms that characterize major depressive disorder (MDD), a mood disorder with a disease burden that rivals that of heart diseases. The use of probiotics to treat depression has gained attention in recent years, as evidenced by increasing numbers of animal and human studies that have supported the antidepressive efficacy of probiotics. Physiological changes observed in these studies allow for the elucidation of probiotics antidepressive mechanisms, which ultimately aim to restore proper functioning of the MGB axis. However, the understanding of mechanisms does not yet complete the endeavor in applying probiotics to treat MDD. Other challenges remain which include the heterogeneous nature of both the gut microbiota composition and depressive symptoms in the clinical setting. Nevertheless, probiotics offer some advantages over standard pharmaceutical antidepressants, in terms of residual symptoms, side effects and stigma involved. This review outlines antidepressive mechanisms of probiotics based on the currently available literature and discusses therapeutic potentials of probiotics for depression.

## Introduction

Approximately 10^14^ microbes, also known as gut microbiota, reside in the human gastrointestinal tract. The majority of these microbes belong to the Firmicutes, Bacteroidetes, Actinobacteria and Proteobacteria phyla. The gut microbiota flourishes in a symbiotic alliance with the host and, as such, has eminent regulatory effects on the host physiology. The gut microbiota actively engages with the proper development and functioning of both the immune system and brain. This is mediated by the microbiota–gut–brain (MGB) axis that lays the foundation for the intricate communicative pathways between gut microbiota and the nervous, immune and endocrine systems. However, the diversity and richness of gut microbiota are susceptible to change based on the host’s lifestyle. An adverse change induces a gut dysbiosis which disrupts the symbiosis maintained by the MGB axis. Indeed, a gut dysbiosis has been linked to various health conditions, such as obesity, IBS, schizophrenia, Parkinson’s disease and MDD (Sherwin et al., 2016; Thursby and Juge, 2017; van de Guchte et al., 2018).

Major depressive disorder is currently the leading cause of disability worldwide and is expected to outrank heart diseases as the number one disease burden by 2030 (Reddy, 2010; Tucci and Moukaddam, 2017). According to the Diagnostic and Statistical Manual of Mental Disorders-5, MDD is diagnosed when a person experiences most of the following symptoms for at least 2 weeks: depressed mood, anhedonia, excessive guilt, suicidal ideation, changes in appetite and sleep, psychomotor retardation, poor concentration and fatigue. Among these criteria, either depressed mood or anhedonia (or both) must be present for a diagnosis of MDD (American Psychiatric Association, 2013). In this review, the term “depression” would be used to refer to symptoms that characterize MDD.

A causal relationship potentially exists between the gut microbiota and MDD. Germ-free (GF) rodents developed depressive-like behaviors following fecal microbiota transplantation from MDD patients, but not from healthy people (Kelly et al., 2016; Zheng et al., 2016). As compared to healthy individuals, MDD patients have a different gut microbiota profile. The decrease in *Faecalibacterium*, *Bifidobacterium*, *Lactobacillus* (Aizawa et al., 2016), and *Dialister* (Kelly et al., 2016), and increase in *Clostridium*, *Streptococcus*, *Klebsiella*, *Oscillibacter*, *Allistipes* (Naseribafrouei et al., 2014; Jiang et al., 2015; Lin et al., 2017; Rong et al., 2019), *Eggerthella*, *Holdemania*, *Gelria*, *Turicibacter*, *Paraprevotella*, and *Anaerofilum* (Kelly et al., 2016) genera have been found among MDD patients. This shift in the gut microbiota composition may contribute to a shift in the regulation of the host physiology (Luan et al., 2017). It is, thus, worthwhile to tackle MDD from the MGB axis standpoint, with an emphasis on the gut microbiota.

Probiotics are microbes (usually lactic acid bacteria such as Lactobacilli and Bifidobacteria) that benefit the host physiology upon ingestion. Probiotics are marketed in the form of capsules, powder or fermented products. The global market size of probiotics amount to billions and is increasing annually due to consumers’ interest in optimizing their health with functional foods (Di Cerbo and Palmieri, 2015). Probiotics have been utilized to modulate the MGB axis in an attempt to treat diseases, including MDD. Meta-analyses and systematic reviews have already supported the efficacy of probiotics in reducing clinical depression and depressive-like symptoms in MDD patients and healthy individuals, respectively (Huang et al., 2016; Pirbaglou et al., 2016; Wang et al., 2016; McKean et al., 2017; Wallace and Milev, 2017).

To what extent are probiotics viable tools to treat MDD/depression? This review addresses this question by first outlining the workings of MGB axis and process by which this axis becomes maladaptive, leading to the development of depression. Antidepressive mechanisms of probiotics are further elucidated by drawing parallels between the physiological outcomes that accompanied the behavioral changes to the MGB axis from animal and human research. Lastly, in light of the heterogeneous nature of both the gut microbiota composition and depression subtypes in the clinical setting, challenges and potentials in translating probiotics for clinical use are discussed.

## The MGB Axis and Depression

### Signaling Pathways of the MGB Axis: Neural and Humoral Routes

The first point of contact between the gut microbiota and host nervous system is likely via the enteric nervous system (ENS). The ENS has been described as “the second brain” due to its neuronal complexity on par with the brain and its ability to function as an independent, discrete unit to regulate gut-related activities and the immune system (Furness, 2012; Breit et al., 2018). Without gut microbiota, the excitability of enteric neurons would likely be attenuated, based on data observed in GF mice (McVey Neufeld et al., 2013). Through the ENS, gut microbiota and the brain communicate bidirectionally through neural and humoral (systemic circulation) pathways (Luan et al., 2017). Parasympathetic vagus afferents carry neural information from internal organs, including the gut, to the brain (Breit et al., 2018). The vagus nerve also consists of motor neurons that innervate nearly all enteric neurons (Powley, 2000). This enables the brain to influence the activity of ENS to some extent, particularly the state of intestinal permeability and gut inflammation. Sympathetic spinal nerves also connect enteric neurons to the brain, albeit to a lesser extent than vagal nerves (Lomax et al., 2010; Breit et al., 2018). Additionally, the humoral route allows microbial metabolites to enter the systemic circulation and exert its effects elsewhere, including the brain. Likewise, the brain also sends chemical messengers, such as cytokines and glucocorticoids, via the humoral route to regulate the gut physiology (Luan et al., 2017).

### Signaling Mechanisms of the MGB Axis: Immune, Endocrine, and Neurotransmitter Systems

The gastrointestinal tract contains approximately 70% of the immune system (Vighi et al., 2008). Immune cells express TLRs that respond to foreign antigens, such as LPS, as they penetrate the intestinal mucosal barrier. This promptly triggers production of inflammatory cytokines, mainly ILs, tumor necrosis factor (TNF)-α and IFN-γ (Sherwin et al., 2016). These cytokines enter the brain through various pathways. The humoral pathway enables cytokines to enter circumventricular organs or permeable regions of the BBB or bind to carrier proteins that cross the BBB. The neural pathway allows gut cytokines to stimulate specific brain areas such as the brainstem, hypothalamus and limbic structures via vagus and spinal afferents. The cellular pathway allows cytokines to be transported into the brain by the action of monocytes or macrophages. These cytokines could also bind to receptors on astrocytes and microglia, and subsequently trigger cytokine production within the brain (Schiepers et al., 2005; Miller and Raison, 2016).

When proinflammatory signals reach the brain, the hypothalamic-pituitary-adrenal (HPA) axis, a sympathetic-neuroendocrine system, is activated to restore homeostasis. In response to stress, the hypothalamic paraventricular nucleus (PVN) synthesizes and releases corticotropin-releasing factor (CRF) to stimulate the anterior pituitary gland to release adrenocorticotropic hormone (ACTH) into the systemic circulation. ACTH stimulates the adrenal cortex to release glucocorticoids (cortisol in humans and corticosterone in rodents) which inhibit the release of CRF, establishing a negative feedback loop. Glucocorticoids are core effectors of the HPA axis that travel by the humoral route to exert its adaptive effects elsewhere; for instance, to reduce gut inflammation (Tsigos and Chrousos, 2002; Schiepers et al., 2005).

Furthermore, neurotransmitters in the brain serve indispensable roles in maintaining proper brain functions. Neurotransmitters such as GABA, glutamate (Glu), serotonin (5-HT), DA, NE, histamine and acetylcholine (ACh) are known to be synthesized by the gut microbiota (Oleskin et al., 2016). Notably, *Lactobacillus*, a prominent probiotic genus, produces multiple neurotransmitters in a species-dependent manner *in vitro* (Table 1). It should be noted that gut-derived neurotransmitters are functionally different from brain-derived neurotransmitters (Mittal et al., 2017). The bioavailability of precursors for these neurotransmitters is also regulated by the gut microbiota. For example, carbohydrate-fermenting microbes secrete butyrate (a SCFA) that stimulates 5-HT synthesis from intestinal enterochromaffin cells (ECs) (Reigstad et al., 2015; Yano et al., 2015; Lund et al., 2018). In contrast, Clostridia metabolites, such as 4-cresol and 4-hydroxyphenylacetate (4-HPA), inhibit dopamine-β-hydroxylase (an enzyme that converts DA to NE in the brain) (Shaw, 2017). These microbial neuroactive molecules likely modulate local ENS signaling, which ultimately influence the MGB axis (Karl et al., 2018).

**TABLE 1:** The neurotransmitters produced by probiotics and their regulatory functions.

**Neurotransmitter**	**Regulatory functions**	**Probiotics**	**References**
Gamma-aminobutyric acid (GABA)	• Hippocampal neurogenesis • HPA axis regulation • Mood	*L. brevis* *L. rhamnosus* *L. reuteri L. paracasei* *L. plantarum L. bulgaricus L. helveticus* *L. casei*	Komatsuzaki et al. (2005), Luscher et al. (2011), Stromeck et al. (2011), Barrett et al. (2012), Liao et al. (2013), Lin (2013), Oleskin et al. (2014), Yunes et al. (2016)
Serotonin (5-HT)	• Impulsivity • Aggression • Appetite • Circadian rhythm • Learning • HPA axis regulation • Mood	*L. plantarum* *L. helveticus*	Özogul (2011), Özoğul et al. (2012), Oleskin et al. (2014), Carhart-Harris and Nutt (2017)
Dopamine (DA)	• Motivation • Concentration • Psychomotor speed • Ability to experience pleasure • Mood	*L. plantarum L. helveticus L. casei L. bulgaricus*	Dunlop and Nemeroff (2007), Özogul (2011), Oleskin et al. (2014)
Norepinephrine (NE)	• Aggression • Cognitive function • Sleep • Sympathetic activity • HPA axis regulation • Mood	*L. helveticus* *L. casei* *L. bulgaricus*	Leonard (2001), Montgomery and Briley (2011), Oleskin et al. (2014)
Glutamate (Glu)	• Gastrointestinal reflexes • Intestinal motility • HPA axis regulation • Mood	*L. rhamnosus* *L. reuteri* *L. plantarum L. paracasei L. helveticus* *L. casei* *L. bulgaricus*	Weingand-Ziadé et al. (2003), Zalán et al. (2009), Stromeck et al. (2011), Zareian et al. (2012), Julio-Pieper et al. (2013), Oleskin et al. (2014)
Histamine	• Motivation • Learning • Memory • Appetite • Sleep • Sympathetic activity • Mood	*L. plantarum* *L. reuteri*	Kano et al. (2004), Özoğul et al. (2012), Thomas et al. (2012), Torrealba et al. (2012), Hemarajata et al. (2013)
Acetylcholine (ACh)	• Cognition • Synaptic plasticity • Analgesia • Sleep • HPA axis regulation • Mood	*L. plantarum*	Rowatt (1948), Girvin and Stevenson (1954), Pytka et al. (2016)

### Dysregulated MGB Axis in Depression: Chronic Stress Response Loop

**FIGURE 1:**
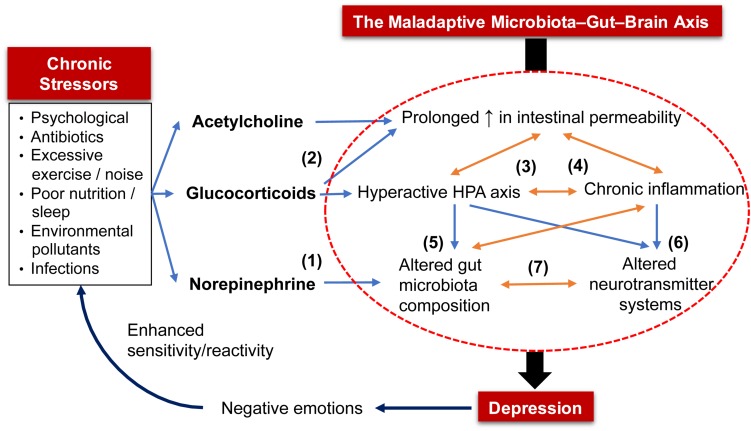
The maladaptive microbiota–gut–brain (MGB) axis in the pathophysiology of depression. Chronic exposure to stressors (e.g., psychological, poor nutrition) triggers prolonged release of (1) norepinephrine that alters gut microbiota composition by shifting to one that is enriched with pathogenic bacteria, and (2) acetylcholine and glucocorticoids that increase intestinal barrier permeability. The increased intestinal permeability allows bacteria and their toxins to enter systemic circulation, triggering stress responses from the HPA axis and immune system that, when excessive; (3) leads to chronic inflammation and HPA axis overactivity; (4) aggravate intestinal permeability; (5) alter composition of gut microbiota; and (6) disrupt neurotransmitter systems. Altered gut microbiota also results in an inflamed gut and (7) a shift in the production of bioactive molecules that regulate host neurotransmitter systems and gut motor functions. As a proof of concept, these five factors (in the circle) that depict the maladaptive MGB axis are often detected in MDD patients. Lastly, the constant negative emotions displayed by depressed patients further trigger a stronger reaction or sensitivity to various stressors.

Acute psychological stress increases the release of ACh from cholinergic nerves (Saunders et al., 1997; Kiliaan et al., 1998) and glucocorticoids from the HPA axis (Alonso et al., 2012; Zheng et al., 2013; Vanuytsel et al., 2014), both of which loosen tight junctions of the intestinal barrier (Figure 1). Other stressors such as poor diet, sleep deprivation, antibiotics, environmental pollutants and excessive exercise also increase the intestinal permeability (Karl et al., 2018). Additionally, exposure to stress stimulates sympathetic spinal nerves to release NE into the gut which expedites quorum sensing systems and iron uptake of bacteria, leading to increased virulence and growth of pathogenic bacteria (e.g., *Escherichia coli*, *Salmonella*, *Campylobacter*, etc.) (Lomax et al., 2010; Freestone, 2013). These factors facilitate penetration of bacteria and their toxins, such as LPS, through the weakened intestinal barrier. Administration of LPS increased proinflammatory cytokines and caused anxiety and depression in healthy males in a dose-dependent manner (Grigoleit et al., 2011). This phenomenon is only transient due to the adaptive response of the immune system and HPA axis. However, chronic stress prevents this homeostatic restoration and causes prolonged inflammation and HPA axis overactivity, both of which aggravate the disrupted intestinal barrier. During this process, chronic inflammation renders the immune system insensitive to inhibitory signals from glucocorticoids (de Punder and Pruimboom, 2015). Excess proinflammatory cytokines, in turn, disrupt the negative feedback inhibition of circulating glucocorticoids of the HPA axis (Schiepers et al., 2005; Miller et al., 2009). Indeed, MDD patients often show increased intestinal barrier permeability (Stevens et al., 2018; Calarge et al., 2019; Ohlsson et al., 2019) and elevated serum antibodies against LPS (Maes et al., 2008).

Excessive glucocorticoids hyperactivate monoamine oxidases (MAOs; enzymes that degrade 5-HT, NE, and DA) (Grunewald et al., 2012). An overactive HPA axis can also induce gut dysbiosis (Murakami et al., 2017) and impairment of brain neurotransmitter systems (Pacak et al., 1993; Smith et al., 1995; Lopez et al., 1998; Hewitt et al., 2009). Higher baseline levels of cortisol, an indicator of an overactive HPA axis, were detected in more than 70% of MDD patients (Vreeburg et al., 2009; Lok et al., 2012). Proinflammatory cytokines and glucocorticoids upregulate indoleamine 2,3-dioxygenase (IDO) and tryptophan-2,3-dioxygenase (TDO) enzymes, respectively (Schimke et al., 1965; Young, 1981). Both enzymes metabolize TRP into KYN and quinolinic acid, which reduce the bioavailability of TRP to cross the BBB, thereby lowering 5-HT synthesis (Reus et al., 2015). This is evidenced by low plasma TRP levels that were also correlated to a heightened proinflammatory state found in MDD patients (Maes et al., 1993, 1994). Furthermore, proinflammatory cytokines can decrease levels of DA, 5-HT and NE in the brain by upregulating their reuptake via presynaptic transporters and downregulating enzymatic cofactors required for their synthesis (Miller and Raison, 2016). Indeed, administration of cytokines consistently induced neurotransmitter imbalances in the brain and behavioral changes that are reminiscent of depression in animals and humans (Miller et al., 2009). Similarly, higher levels of proinflammatory cytokines were observed in depressed individuals as reported using meta-analyses of the data available in the literature (Howren et al., 2009; Dowlati et al., 2010).

A stress-induced inflamed gut adversely alters the relative abundances of preexisting bacteria in the gut (Figure 1). Acute psychological stress stimulated the release of inflammatory mediators that were correlated with the lowered abundance of *Coprococcus*, *Pseudobutyrivibrio, Dorea*, and *Lactobacillus* in mice. This, in turn, allowed the proliferation of *Clostridium* species in the gut (Bailey et al., 2011). The gut microbiota of chronic-stressed mice also deviated from the baseline, whereby an increase in proinflammatory bacteria, such as *Helicobacter* and *Streptococcus*, and a decrease in butyrate-producing bacteria, such as *Roseburia* and *Lachnospiraceae* species, were observed (Gao et al., 2018). Altered gut microbiota composition consequently exacerbates gut inflammation and further increases intestinal permeability and production of proinflammatory cytokines (van de Guchte et al., 2018). The precise mechanism underlying vulnerability of certain bacteria to inflammation remains poorly understood. It is hypothesized that inflammation disrupts β-oxidation of intestinal epithelial cells (IECs, both enterocytes and colonocytes) to increase oxygen content in the gut lumen. This promotes formate oxidation that favors the growth of facultative anaerobes, such as *E. coli*, that are pathogenic and inflammatory at the cost of obligate anaerobes, such as Bacteroides and Firmicutes (Hughes et al., 2017).

A dysregulated gut microbiota translates to a shift in the production of neuroactive metabolites and alters host neurotransmitter circuitry. This corresponds with disrupted levels of neurotransmitters in the brain of GF mice (Diaz Heijtz et al., 2011; Neufeld et al., 2011; Clarke et al., 2013; Pan et al., 2019). Altered neurotransmitter profile (e.g., GABA, Glu, 5-HT, DA, and NE) has been associated with the pathophysiology of depression. Therefore, pharmaceutical antidepressants function to restore synaptic levels of neurotransmitters (Harald and Gordon, 2012). In addition, impaired neurotransmitter systems within the ENS may alter gut motor function. This has direct implications as gut motility is a determining factor in the size and diversity of gut microbiota (Quigley, 2011). Therefore, chronic stress sets up a vicious cycle of increased intestinal permeability, chronic inflammation, hyperactive HPA axis, altered gut microbiota profile and neurotransmitter imbalances – forming a maladaptive MGB axis (Figure 1). Furthermore, MDD patients perceive stress as more threatening and challenging to cope with compared to healthy individuals (Farabaugh et al., 2004; Salomon et al., 2009). These negative emotions can increase their sensitivity to stressors, such as an elevated cortisol response (Mendonca-de-Souza et al., 2007). To restore this malfunctioned axis, probiotics have been demonstrated by meta-analyses and systematic reviews as a potential treatment for MDD/depression (Huang et al., 2016; Pirbaglou et al., 2016; Wang et al., 2016; McKean et al., 2017; Wallace and Milev, 2017). Potential antidepressive mechanisms of probiotics are elucidated in the following section.

## Delineating the Antidepressive Mechanisms of Probiotics

**FIGURE 2:**
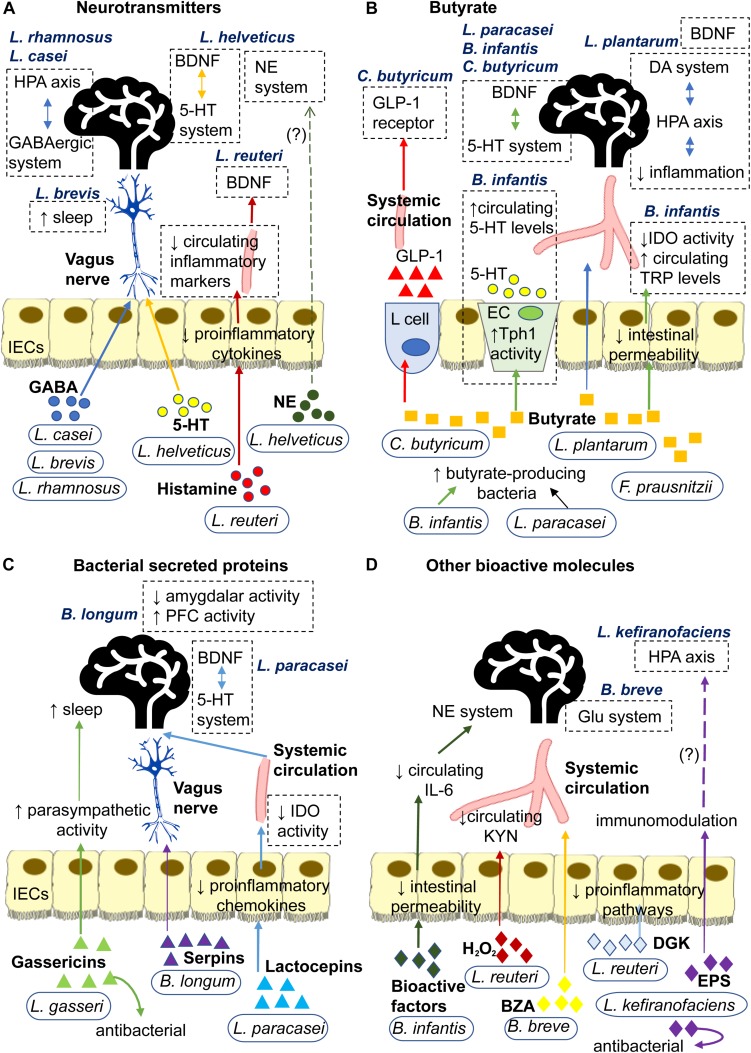
Signaling mechanisms underlying antidepressive effects of probiotics mediated through secretion of **(A)** Neurotransmitters: *L. rhamnosus* and *L. casei* secrete GABA that may signal central GABAergic system and HPA axis via the neural route. *L. brevis* secretes GABA that enhances sleep. *L. helveticus* secrete 5-HT that may signal the central 5-HT system via the neural route. *L helveticus* also secretes NE that may affect the central NE system. *L. reuteri* secretes histamine that decreases secretion of proinflammatory cytokines by IECs. This may reduce circulating inflammatory markers, such as LPS, IL-6 and corticosterone, and subsequently prevent the inflammation-induced decrease in hippocampal BDNF. **(B)** Butyrate: *L. plantarum* produces butyrate that strengthens intestinal barrier and diffuses through the circulation to regulate BDNF expression and reduce inflammation in the brain. The latter consequently regulates the HPA axis and its regulator, the DA system. *C. butyricum* produces butyrate that influences central 5-HT and BDNF systems and stimulates L cell to secrete GLP-1 into the bloodstream which increases expression of GLP-1 receptors. *F. prausnitzii* produces butyrate that strengthens the intestinal barrier. *B. infantis* and *L. paracasei* promote growth of butyrate-producing bacteria. Through butyrate, *B. infantis* upregulates Tph1 activity of EC which increases circulating 5-HT and strengthens intestinal barrier to lower IDO activity and increase circulating TRP, both of which affect the central 5-HT system and BDNF expression. Through butyrate, *L. paracasei* may influence the central 5-HT system and BDNF expression. **(C)** Bacterial secreted proteins: *L. gasseri* secretes gassericins that increase parasympathetic activity to facilitate sleep and improves gut microbiota composition. *B. longum* secretes serpins that alter neural activities in the brain via the neural route. *L. paracasei* secretes lactocepins that decrease proinflammatory chemokines in IECs. This lowers IDO activity which, in turn, affects the central 5-HT system and BDNF expression. **(D)** Other bioactive molecules: *B. infantis* secretes bioactive factors (likely polysaccharides) that decrease circulating IL-6 which affects the central NE system. *L. reuteri* secretes H_2_O_2_ that decreases IDO activity and circulating KYN, and dgk that inhibits the initiation of proinflammatory pathways. *B. breve* converts albiflorin into BZA which affects the Glu system via the humoral route. *L. kefiranofaciens* secretes exopolysaccharides that have immunomodulatory and antibacterial properties, which may potentially prevent HPA axis overactivity. 5-HT, 5-hydroxytryptamine or serotonin; BDNF, brain-derived neurotrophic factor; DA, dopamine; BZA, benzoic acids; dgk, diacylglycerol kinase; ECs, enterochromaffin cells; EPS, exopolysaccharide; GABA, gamma-Aminobutyric acid; GLP-1, glucagon-like peptide-1; Glu, glutamate or glutaminergic; H_2_O_2_, hydrogen peroxide; HPA, hypothalamic-pituitary-adrenal; IECs, intestinal epithelial cells; IDO, indoleamine 2,3-dioxygenase; IL-6, interleukin-6; KYN, kynurenine; NE, norepinephrine; LPS, lipopolysaccharides; Tph1, tryptophan hydroxylase 1; TRP, tryptophan.

Probiotics secrete a wide range of signaling molecules that operate via distinct pathways to exert their effects, be it antidepressive, immunomodulatory or modulation of neurotransmission (Luan et al., 2017). This review classifies probiotic-associated signaling molecules into four types: neurotransmitters, bacterial secreted proteins, butyrate and other bioactive molecules (Figure 2). Some probiotics can secrete signaling molecules of different types. In this regard, the mechanisms of individual probiotics will be presented in the order of pertinence and similarity to each other.

### Lactobacillus rhamnosus

*Lactobacillus rhamnosus* JB-1, the typical experimental strain of *L. rhamnosus*, was formerly referred to as *Lactobacillus reuteri*. Orally administered *L. rhamnosus* reduced depressive-like behaviors in normal, healthy mice (Bravo et al., 2011) and chronic-stressed mice (McVey Neufeld et al., 2018). Postpartum women (Slykerman et al., 2017) and obese individuals (Sanchez et al., 2017) that were supplemented with *L. rhamnosus* reported lower depressive thoughts compared to the control group. In vagotomized rats, behavioral and physiological benefits of *L. rhamnosus* were abolished (Bravo et al., 2011). This substantiates the vagus nerve as an essential conduit in the signaling pathway of *L. rhamnosus*. Introduction of *L. rhamnosus* into the gut lumen heightened the firing rate of vagus nerve and enteric neurons in mice (Perez-Burgos et al., 2013, 2014). These findings suggest that *L. rhamnosus* signals to the brain via the neural route, which may influence the central GABAergic system and HPA axis to manifest an antidepressive effect (Figure 2A). However, it is unclear whether neurotransmitters, cytokines or other molecules are involved in the neural signaling of *L. rhamnosus*.

#### Microbial GABA, Central GABAergic System, and HPA Axis

Glutamine is a precursor to Glu while Glu is a precursor to GABA. Reduced levels of GABA and Glx (Glu + glutamine) have been consistently reported in cortical regions of MDD patients (Sanacora et al., 1999; Hasler et al., 2007; Bhagwagar et al., 2008; Moriguchi et al., 2018; Godlewska et al., 2019). A dysfunctional glutaminergic system, that is partly responsible by a decreased GABAergic tone, is also implicated in MDD (Murrough et al., 2017). *N*-acetyl aspartate (NAA) is regarded as a marker for neuronal vitality. In MDD patients, decreased NAA levels in the PFC and hippocampus have been detected (Gonul et al., 2006; Olvera et al., 2010; Lefebvre et al., 2017). These neurochemical (i.e., Glx, NAA, and GABA) levels in the PFC and hippocampus of mice increased when administered with *L. rhamnosus* (Janik et al., 2016), implicating its antidepressive potential.

Intake of *L. rhamnosus* altered the central mRNA expression of GABA_A_ and GABA_B_ receptors while reducing depressive- and anxiety-like behaviors in mice. These effects were also dependent on an intact vagus nerve (Bravo et al., 2011). With prebiotics, *L. rhamnosus* intake decreased hippocampal GABA_Aα2_ mRNA expression in stressed mice (McVey Neufeld et al., 2017). *L. rhamnosus* produced GABA and Glu efficiently from microbial glutamate decarboxylase and glutaminase, respectively, *in vitro* (Stromeck et al., 2011; Liao et al., 2013; Lin, 2013). These biosynthetic machineries utilized by microbes to synthesize Glu and GABA are mutual in neurons (Mathews and Diamond, 2003), which support the interkingdom communication of microbial GABA (Lyte, 2011). It was demonstrated *in vitro* that gut microbial GABA can cross the intestinal barrier via H^+^/GABA symporter (Thwaites et al., 2000; Nielsen et al., 2012). The microbial GABA may subsequently interact with GABA receptors and transporters that are widely expressed on enteric neurons and vagus afferents (Hyland and Cryan, 2010).

Administration of *L. rhamnosus* reduced stress-induced plasma corticosterone levels in mice that averted depression (Bravo et al., 2011; McVey Neufeld et al., 2018). This could be due to the innervation of PVN neurons by GABAergic synapses that can be desensitized by acute stress (Hewitt et al., 2009). Inhibited GABA signals allow continuous release of CRF by PVN neurons, which ultimately leads to cortisol overproduction and HPA axis overactivity (Cullinan et al., 2008). Impairment of GABA receptors also inhibits hippocampal neurogenesis, which has been shown to activate the HPA axis and induce depression in mice (Earnheart et al., 2007; Schloesser et al., 2009). Such effects may be possibly prevented by the production of GABA by *L. rhamnosus*.

### *Lactobacillus casei* Strain Shirota

Individuals with low mood reported feeling happier after consuming milk containing *L. casei*, but not the placebo (Benton et al., 2007). Intake of mixed-species probiotics that included *L. casei* also reduced clinical depression and depressive-like symptoms in MDD patients (Akkasheh et al., 2016) and healthy individuals (Steenbergen et al., 2015; Mohammadi et al., 2016), respectively. Similar to *L. rhamnosus*, evidence suggests that *L. casei* may also regulate the HPA axis via the neural route (Figure 2A).

#### Microbial GABA and HPA Axis

Intake of *L. casei* stimulated vagus afferents and decreased both the activity and quantity of CRF-expressing cells in PVN of rats (Takada et al., 2016). Intragastric injection of *L. casei* downregulated the activity of sympathetic efferents to adrenal glands and liver, and this effect ceased upon vagotomy (Tanida et al., 2014). In clinical trials, *L. casei* supplementation lowered salivary cortisol levels, feelings of stress and frequency of abdominal- and flu-related symptoms in stressed individuals (Kato-Kataoka et al., 2016; Takada et al., 2016). These studies imply that *L. casei* prevents HPA axis overactivity via the vagus nerve, which may consequently lower stress-related feelings and illnesses. *L. casei* produced GABA *in vitro* (Oleskin et al., 2014), indicating a possibility that it may share an antidepressive mechanism of *L. rhamnosus*. Stressed individuals that consumed *L. casei* showed improvements in mental health and gut microbiota composition, characterized by increased *Lactobacillus* and *Bifidobacterium* populations (Rao et al., 2009; Kato-Kataoka et al., 2016). As most of the antidepressive probiotics belong to *Lactobacillus* and *Bifidobacterium* genera, the potential antidepressive capacity of *L. casei* is highly supported.

### Lactobacillus brevis

Similar to *L. rhamnosus* and *L. casei*, *L. brevis* produces GABA via glutamate decarboxylase in substantial amounts (Yokoyama et al., 2002; Siragusa et al., 2007; Barrett et al., 2012; Ko et al., 2013; Yunes et al., 2016). This indicates that *L. brevis* may share a mutual mechanism of action with *L. rhamnosus* and *L. casei* (Figure 2A). Although *L. brevis* has been shown to influence neither the central GABAergic system nor the HPA axis, *L. brevis* appears to promote sleep.

#### Microbial GABA and Sleep

Milk fermented with *L. brevis* had increased GABA content. This *L. brevis*-fermented milk demonstrated an antidepressive potency on par with fluoxetine, a SSRI, in depressed rats (Ko et al., 2013). Intriguingly, intake of *L. brevis*-produced GABA improved sleep duration in mice (Han et al., 2017). Another study also showed that dietary *L. brevis* enhanced sleep quality and voluntary physical activity in mice (Miyazaki et al., 2014). GABA is the main inhibitory neurotransmitter that is widely associated with sleep, and GABA receptors are frequent targets for pharmaceutical drugs, such as benzodiazepine, to treat insomnia (Gottesmann, 2002). GABA-enriched foods and GABA extract have also been shown to improve sleep quality in insomniacs (Byun et al., 2018) and healthy individuals (Yamatsu et al., 2015). Therefore, *L. brevis* has therapeutic value for insomnia, which reflects one of the diagnostic criteria for MDD (American Psychiatric Association, 2013).

### Lactobacillus reuteri

Treatment of *L. reuteri* ameliorated depressive-like behaviors in chronic-stressed (Marin et al., 2017) and immobilization-stressed mice (Jang et al., 2019). The former study further elucidated the mechanism of *L. reuteri* which involves regulation of IDO, a rate-limiting enzyme of immune cells that catabolizes TRP to KYN (Reus et al., 2015). It is also well documented that *L. reuteri* exhibits anti-inflammatory activities (Thomas et al., 2012; Gao et al., 2015; Ganesh et al., 2018). It is, thus, conceivable that *L. reuteri* may also prevent activation of IDO by proinflammatory cytokines (Reus et al., 2015).

#### Microbial Hydrogen Peroxide and Kynurenine Pathway

The etiology of depression is partly attributed to a dysregulated KYN/TRP pathway (Reus et al., 2015). An elevated ratio of plasma KYN/TRP often correlates positively with the depression severity in human (Maes et al., 2002; Gabbay et al., 2010; Baranyi et al., 2013; Zhou et al., 2019). It was demonstrated that *L. reuteri* intake improved behaviors of depressed mice by reversing the stress-induced (1) decrease in fecal H_2_O_2_ levels and *Lactobacillus* populations, and (2) increase in intestinal IDO1 expression and plasma KYN levels (Marin et al., 2017). KYN administration attenuated this antidepressive effect, which indicates that *L. reuteri* ameliorates depression by reducing plasma KYN levels. This study also showed that *L. reuteri* generated high amounts of H_2_O_2_ *in vitro*, and the author proposed that H_2_O_2_ is the key metabolite in mediating antidepressive effect of *L. reuteri* (Marin et al., 2017). This is because H_2_O_2_ catalyzes peroxidase-mediated reactions that inhibit IDO activity (Freewan et al., 2013). H_2_O_2_ is transported by aquaporin-3 transporters that are expressed on IECs (Thiagarajah et al., 2017) and immune cells (Moon et al., 2004). These findings suggest that microbial H_2_O_2_ can potentially cross the intestinal barrier to suppress IDO activity in immune cells, which would lower circulating KYN levels (Figure 2D).

#### Microbial Histamine, Diacylglycerol Kinase, and Brain-Derived Neurotrophic Factor (BDNF) Expression

*Lactobacillus reuteri* possesses histidine decarboxylase that converts dietary L-histidine to histamine, which inhibits the production of TNF-α *in vitro* (Thomas et al., 2012; Hemarajata et al., 2013). The microbial histamine suppressed proinflammatory cytokine activities in IECs via the histamine-2 receptor signaling pathway in mice. This effect disappeared when the histidine decarboxylase gene of *L. reuteri* was inactivated by mutagenesis (Gao et al., 2015). Intriguingly, microbial histamine also activated histamine-1 receptors to initiate downstream proinflammatory pathways in mice (Ganesh et al., 2018). However, the substrate for this pathway, diacylglycerol, is metabolized to phosphatidic acid by diacylglycerol kinase produced by *L. reuteri*. Thus, *L. reuteri* secretes both histamine and diacylglycerol kinase that act on histamine receptors to produce an anti-inflammatory effect (Ganesh et al., 2018). Orally administered *L. reuteri* simultaneously alleviated colitis and behaviors indicative of anxiety and depression in stressed mice. These effects were also accompanied by a decrease in colon inflammation and blood levels of LPS, interleukin-6 (IL-6) and corticosterone. In the same study, this reduction in peripheral inflammation prevented the infiltration of activated microglia into the hippocampus and increased hippocampal BDNF expression (Jang et al., 2019; Figure 2A). BDNF has been extensively studied for its vital role in neuronal function and its causal link to depression. Antidepressants such as SSRI and ketamine also increase hippocampal BDNF expression as part of their mechanism of action (Bjorkholm and Monteggia, 2016). Furthermore, this anti-inflammatory effect of *L. reuteri* may prevent IDO activation by proinflammatory cytokines (Reus et al., 2015).

### Lactobacillus plantarum

*Lactobacillus plantarum* supplementation decreased depressive-like symptoms in chronic-stressed mice (Liu Y.W. et al., 2016; Dhaliwal et al., 2018) and stressed adults with mild depression (Lew et al., 2018), though the latter study did not reach statistical significance. Following *L. plantarum* intake, reduction in plasma corticosterone levels and inflammation were seen in mice with reduced depressive-like behaviors (Liu Y.W. et al., 2016). Another study reported that mice fed with *L. plantarum* displayed an increase in cecum SCFAs levels (acetic and butyric), and a decrease in intestinal permeability and level of MAOs in the brain (Dhaliwal et al., 2018). These physiological changes can be unified into a mutual mechanism that *L. plantarum* likely mitigates systemic inflammation (Figure 2B).

#### Butyrate, Intestinal Barrier, and BDNF Expression

Chronic-stressed mice fed with *L. plantarum* exhibited reduced depressive-like behaviors, coupled with an increase in butyrate and butyrate-producing bacteria, such as *Lactobacillus*, *Bacteroidetes*, and *Roseburia* (Dhaliwal et al., 2018). *L. plantarum* synthesizes butyrate via fatty acid synthase II–thioesterase, a glutamine-mediated butyrogenic pathway (Botta et al., 2017). Butyrate can enter IECs through cholesterol-rich microdomains and/or monocarboxylate transporter 1 protein (Suzuki et al., 2008; Goncalves et al., 2011; Nedjadi et al., 2014), and promote synthesis and assembly of tight junction proteins of IECs (Bordin et al., 2004; Ohata et al., 2005; Peng et al., 2009; Wang et al., 2012; Yan and Ajuwon, 2017). Butyrate also has anti-inflammatory properties; for instance, butyrate inhibited proinflammatory activities of IECs *in vitro* (Elce et al., 2017) and interacted with IECs to regulate host T cell responses (Lew et al., 2018; Xu et al., 2018). Butyrate may also diffuse into the systemic circulation to exert anti-inflammatory effects on various organs and tissues, including the brain (McNabney and Henagan, 2017; Matt et al., 2018). Indeed, butyrate has been shown to normalize behavior of depressed rodents through epigenetic regulations of hippocampal BDNF expression (Han et al., 2014; Wei et al., 2014; Sun et al., 2016). These outcomes are consistent with the finding that *L. plantarum* intake increased hippocampal BDNF expression and cecum butyrate levels in chronic stress-induced depressed mice (Dhaliwal et al., 2018).

#### HPA Axis and Central DA System

*Lactobacillus plantarum* supplementation decreased MAOs levels in brain tissues of mice with reduced depression (Dhaliwal et al., 2018). This is in line with another finding that *L. plantarum* intake in mice increased levels of DA and its metabolites (HVA and 3,4-dihydroxyphenylacetic acid, DOPAC) in the PFC, along with reduced depressive-like behaviors (Liu Y.W. et al., 2016). However, another study showed that *L. plantarum* increased DA levels in the striatum of mice while alleviating anxiety-like behaviors (Liu W.H. et al., 2016). These studies suggest that *L. plantarum* likely affects the central DA system in a context-dependent manner. It was also proposed that *L. plantarum* increases DA levels in the PFC to prevent HPA axis overactivation (Liu Y.W. et al., 2016). DA neurons in the PFC and ventral tegmental area (VTA) form the mesocortical pathway which regulates reward-seeking behaviors (Pariyadath et al., 2016) and the HPA axis (Sullivan and Dufresne, 2006). Glucocorticoids from the HPA axis can also influence the DA system either directly or indirectly, via epigenetic control and MAOs inhibition, respectively (Feenstra et al., 1992; Grunewald et al., 2012; Butts and Phillips, 2013). Taken together, *L. plantarum* may regulate both the DA system and HPA axis by attenuating glucocorticoid-induced MAOs activity.

### *Faecalibacterium prausnitzii* (Previously Known as *Fusobacterium prausnitzii*)

Recently, it was discovered that oral gavage of *F. prausnitzii* exerted antidepressive and anxiolytic effects in chronic-stressed mice (Hao et al., 2019). *F. prausnitzii*, as the sole species of *Faecalibacterium* genera (Duncan, 2002), represents around 5% of the total human gut microbiota (Hold et al., 2003). Low populations of *F. prausnitzii* correlated with the disease severity of those with MDD (Jiang et al., 2015) and bipolar depression (Evans et al., 2017). In a recent large cohort study, fecal levels of *F. prausnitzii* correlated negatively with depressed mood and positively with quality of life (Valles-Colomer et al., 2019). Therefore, *F. prausnitzii* seems to have pertinent contributions to mental health.

#### Butyrate, Microbial Anti-inflammatory Molecules, and Peripheral Inflammation

*Faecalibacterium prausnitzii* produces butyrate in large quantities from fermenting glucose and fiber (Duncan, 2002; Hold et al., 2003). *F. prausnitzii* also secretes microbial anti-inflammatory molecules that suppress the proinflammatory nuclear factor (NF)-κB pathway in IECs (Sokol et al., 2008; Quevrain et al., 2016a, b). These immunomodulatory effects are consistent with neurochemical changes observed in *F. prausnitzii*-treated depressed mice, whereby cecum SCFAs and plasma IL-10 levels increased, while corticosterone and IL-6 levels decreased (Hao et al., 2019). Moreover, intragastric administration of *F. prausnitzii* decreased colonic cytokine levels and intestinal permeability in mice with colitis (Laval et al., 2015; Martin et al., 2015). Thus, butyrate produced by *F. prausnitzii* potentially strengthens the intestinal barrier (similar to *L. plantarum*; Figure 2B). However, whether local immunomodulatory effects of *F. prausnitzii* extend to the brain remains unknown. Nevertheless, the ability of *F. prausnitzii* to attenuate gut inflammation is sufficient to reduce depressive- and anxiety-like behaviors in mice (Hao et al., 2019).

### Lactobacillus helveticus

*Lactobacillus helveticus* intake enabled the recovery of chronic- and subchronic-stressed rodents from their state of depression (Liang et al., 2015; Maehata et al., 2019). Probiotic sticks containing *L. helveticus*, in addition to *Bifidobacterium longum*, reduced clinical depression and depressive-like symptoms in MDD patients (Kazemi et al., 2019) and healthy individuals (Messaoudi et al., 2011), respectively. Most of the animal and human studies also showed that *L. helveticus* intake enhanced memory and, sometimes, attention and learning (Ohland et al., 2013; Chung et al., 2014; Luo et al., 2014; Liang et al., 2015; Ohsawa et al., 2018). Cognitive impairments, such as poor memory and concentration, represent one major cluster of MDD symptoms (Sharpley and Bitsika, 2014). Evidence suggests that *L. helveticus* may modulate the central NE system and HPA axis to improve cognition, and the central 5-HT system and BDNF expression to reduce depression (Liang et al., 2015) (Figure 2A).

#### Microbial NE, Central NE System, and HPA Axis

Supplementation of *L. helveticus* improved memory and cognitive performance in chronic-stressed rats, comparable to the SSRI citalopram-treated rats. This memory improvement correlated with increased plasma IL-10 and hippocampal NE levels, and reduced plasma corticosterone and ACTH levels (Liang et al., 2015). A previous study also showed that ingestion of *L. helveticus* enhanced memory and mitigated gut inflammation in neuroinflammation-induced rats (Luo et al., 2014). However, another study reported that memory improvement in *L. helveticus*-treated mice did not correlate with the state of gut inflammation (Ohland et al., 2013). Despite this discrepancy, it is well established that the hippocampal NE system and HPA axis both interact to regulate hippocampal glucose metabolism for memory consolidation (Osborne et al., 2015). This mechanism may be affected by microbial NE as *L. helveticus* produced NE *in vitro* in amounts that exceed the human bloodstream (Oleskin et al., 2014). It was also shown *in vivo* that gut bacteria are responsible for converting conjugated NE into its biologically active form (Asano et al., 2012). This neuroactive NE likely influences the MGB axis, but the exact mechanism remains unknown (Lyte, 2011).

#### Microbial 5-HT and Central 5-HT-BDNF System

Liang et al. (2015) showed that elevated hippocampal 5-HT levels correlated with reduced depression severity in *L. helveticus*-fed rats. The same study also demonstrated that treatment with SSRI citalopram alleviated depression and increased hippocampal BDNF expression and 5-HT levels (Liang et al., 2015). Hence, the antidepressive mechanism appears similar between *L. helveticus* and citalopram. Cultures of *L. helveticus* produced 5-HT at concentrations close to that in the human bloodstream (Oleskin et al., 2014). As shown *in vivo*, the gut microbiota has an indispensable function in deconjugating glucuronide-conjugated 5-HT to generate their free, biologically active counterparts in considerable amounts (Hata et al., 2017). It is hypothesized that gut luminal 5-HT may sensitize 5-HT 3A receptors of enteric neurons by stimulating the glial cell-derived neurotrophic factor of IECs (Hata et al., 2017). 5-HT3 receptors are also expressed on IECs (Hasler, 2009) and vagal afferents (Hillsley and Grundy, 1998). Therefore, it can be speculated that *L. helveticus* influences the central 5-HT circuitry via the neural route. This is supported by a recent study showing that *L. helveticus* intake increased expression of 5-HT 1A receptors in the nucleus accumbens while restoring behaviors of depressed mice (Maehata et al., 2019).

Chronic-stressed mice that ingested *L. helveticus* displayed an increase in hippocampal BDNF levels (Liang et al., 2015) and neurogenesis in the nucleus accumbens (Maehata et al., 2019). Nucleus accumbens is a brain region implicated in reward behavior. The central BDNF and 5-HT systems are synergistic, whereby 5-HT upregulates hippocampal BDNF–TrkB signaling to increase expression and synthesis of BDNF. The elevated BDNF, in turn, facilitates neurogenesis of 5-HT neurons (Martinowich and Lu, 2008; Bjorkholm and Monteggia, 2016). Therefore, *L. helveticus* likely increases hippocampal BDNF levels via modulation of 5-HT circuitry, in a similar manner to SSRIs (Liang et al., 2015).

### Lactobacillus paracasei

Dietary intervention of heat-killed *L. paracasei* prevented mood deterioration in times of stress in healthy individuals (Murata et al., 2018). In corticosterone-induced depressed mice, oral gavage of either live or heat-killed *L. paracasei* exhibited antidepressive efficacy equivalent to or better than fluoxetine. The same study also showed that live and heat-killed *L. paracasei* operated via different mechanisms. Live *L. paracasei* increased 5-HT levels whereas heat-killed *L. paracasei* increased DA levels in the brain (Wei et al., 2019). The signaling mechanism of *L. paracasei* appears independent of the HPA axis (Wei et al., 2019) or vagus afferents (Tanida and Nagai, 2011). The remaining evidence suggests that *L. paracasei* potentially functions via an immune-mediated humoral pathway.

#### Lactocepin, Butyrate, and Central 5-HT-BDNF System

*Lactobacillus paracasei* secretes lactocepin, a *PrtP*-encoded serine protease, that selectively degrades proinflammatory chemokines in inflamed ileal tissue of mice (von Schillde et al., 2012). Lactocepin is most likely a heat-labile cell surface protein unique to *L. paracasei* (Hoermannsperger et al., 2009; von Schillde et al., 2012). Mice fed with live *L. paracasei* exhibited lower inflammatory markers in serum, such as increased IL-10 and glutathione peroxidase and decreased TNF-α and MCP-1 (Huang et al., 2018). Another study showed that oral gavage of live *L. paracasei* with its bacterial products prevented adverse effect of stress on intestinal permeability in rats (Eutamene et al., 2007). This can be linked to a suppressed IDO activity, resulting in higher TRP bioavailability for 5-HT synthesis in the brain (Reus et al., 2015). Following this, it was shown that live *L. paracasei* delivered via gavage increased 5-HT and 5-HIAA (the main metabolite of 5-HT) levels in the hippocampus and striatum of mice (Huang et al., 2018; Wei et al., 2019). As 5-HT facilitates BDNF synthesis (Martinowich and Lu, 2008), the upregulated central 5-HT expression presumably explains the accompanying increase in hippocampal BDNF expression of mice alleviated of depression from *L. paracasei* intake (Wei et al., 2019). Therefore, *L. paracasei* may upregulate the central 5-HT-BDNF system (similar to *L. helveticus*; Figure 2C).

Treatment of live *L. paracasei* also increased fecal *Bifidobacterium* populations while normalizing behaviors of depressed mice (Wei et al., 2019). The gut microbiota profile, inflammatory markers and levels of acetate and butyrate were improved in IBS patients supplemented with live *L. paracasei* (Bertani et al., 2017; Cremon et al., 2018). Reduction in systemic inflammation, coupled with an improvement in hippocampal function, was also observed in obese rats fed with live *L. paracasei* (Chunchai et al., 2018). Thus, live *L. paracasei* may facilitate the colonization of butyrate-producing bacteria to reduce systemic inflammation (similar to *L. plantarum*) and increase 5-HT secretion from ECs (similar to *Bifidobacterium infantis*; Figure 2B).

### Bifidobacterium infantis

In naïve rats, intake of *B. infantis* was shown to alter depression-related biomarkers (Desbonnet et al., 2008). The same group later showed that chronic-stressed mice no longer displayed depressive-like behaviors after *B. infantis* intake (Desbonnet et al., 2010). In flood victims with IBS, *B. infantis* consumption did not affect their IBS symptoms but improved their mental health instead (Murata et al., 2018). *B. infantis* did not influence corticosterone levels in mice (Desbonnet et al., 2008, 2010), implying that the effect of *B. infantis* is likely to be independent of the HPA axis. Evidence suggests that *B. infantis* has immunomodulatory effects that regulate the central NE system (Desbonnet et al., 2010). A recent study also provided support for the antidepressive mechanism of *B. infantis* that involves the hippocampal 5-HT system (Tian et al., 2019).

#### Bioactive Factors, IL-6, and Central NE System

*Bifidobacterium infantis* treatment manifested two physiological changes *in vivo*. First, *B. infantis* decreased plasma IL-6 levels in mice (Desbonnet et al., 2008, 2010) and patients with inflammatory conditions (Groeger et al., 2013). In depressed mice, the IL-6 release also correlated positively with the severity of depression (Desbonnet et al., 2010). Second, *B. infantis* increased NE levels in the murine brainstem (Desbonnet et al., 2010) containing the majority of NE neurons (Schwarz and Luo, 2015). Therefore, *B. infantis* likely regulates plasma IL-6 and central NE system to exert an antidepressive effect.

*Bifidobacterium infantis* secretes bioactive factors (probably polysaccharides) that enhance transepithelial resistance of IECs (Ewaschuk et al., 2008). Other studies involving rodents also showed that *B. infantis* treatment enhanced the intestinal barrier by strengthening the formation of tight junction proteins and anti-inflammatory activities of immune cells (Lomasney et al., 2014; Zuo et al., 2014; Javed et al., 2016). Indeed, bacterial DNA translocation from the gut lumen into the circulation was reduced in *B. infantis*-fed rodents (Osman et al., 2006; Gómez-Hurtado et al., 2012). Bacterial DNA is a potent inducer of TLRs which facilitate the release of proinflammatory cytokines, including IL-6 (Gutierrez et al., 2016). Administration of IL-6 induced depression in mice, and this outcome was prevented by pharmaceutical blockage of NE neurons in the brainstem (Kurosawa et al., 2016). Hence, *B. infantis* potentially modulates the NE system via an immune-mediated humoral route to reduce depression (Figure 2D). This mechanism appears to be independent of the vagus nerve as oral gavage of *B. infantis* also decreased proinflammatory cytokine (including IL-6) levels in vagotomized mice with an inflamed colon (van der Kleij et al., 2008).

#### Butyrate, TRP, and Central 5-HT-BDNF System

Treatment of *B. infantis* upregulated mRNA expression of Tph1 in RIN14B cells, a cell line that mimics ECs (Tian et al., 2019). Tph1 converts TRP to 5-hydroxytryptophan (5-HTP) and aromatic amino acid decarboxylase subsequently converts 5-HTP to 5-HT. *B. infantis*-fed mice displayed reduced depressive-like behaviors, along with an increase in TRP biosynthesis and hippocampal 5-HT and 5-HTP levels. In the same study, *B. infantis* increased cecum butyrate levels and the abundance of butyrate-producing *Bifidobacterium*. The elevated butyrate levels also correlated with increased hippocampal 5-HTP and PFC BDNF levels (Tian et al., 2019). This could be due to the ability of butyrate and other SCFAs to increase Tph1 activity of ECs, thereby promoting 5-HTP and 5-HT secretions (Reigstad et al., 2015; Yano et al., 2015; Lund et al., 2018). This is consequential as ECs contribute about 95% of the bodily 5-HT (El-Merahbi et al., 2015), and that mice with a gut microbiota had 2.8-fold higher plasma 5-HT levels than GF mice (Wikoff et al., 2009). The evidence for the ability of 5-HT to cross the BBB is conflicting (Brust et al., 2000; Wakayama et al., 2002; Nakatani et al., 2008; El-Merahbi et al., 2015). In contrast, 5-HTP readily crosses the BBB and can be converted into 5-HT. Therapeutic 5-HTP has also been shown to treat clinical depression with a potency equivalent to or better than SSRIs (Birdsall, 1998; Jangid et al., 2013; Jacobsen et al., 2016).

Furthermore, *B. infantis* intake increased plasma TRP levels in healthy rats (Desbonnet et al., 2008), but another study with chronic-stressed rats reported otherwise (Desbonnet et al., 2010). The author then suggested that *B. infantis* regulates TRP metabolism differently, depending on the rat strain (Desbonnet et al., 2010). Therapeutic TRP can improve symptoms of mood, sleep and cognitive disorders as TRP readily passes through BBB to regulate numerous brain functions, such as 5-HT synthesis (Richard et al., 2009). The elevated plasma TRP levels from *B. infantis* intake is most likely a result of reduced proinflammatory cytokines (Desbonnet et al., 2008, 2010), which reduces IDO activity and prevents over-catabolism of TRP (Reus et al., 2015). Thus, *B. infantis* may upregulate the hippocampal 5-HT system via modulation of peripheral 5-HTP, 5-HT and/or TRP levels. As 5-HT promotes BDNF synthesis (Martinowich and Lu, 2008), this presumably explains the concomitant increase in BDNF levels in PFC of rats ameliorated of depression with *B. infantis* treatment (Tian et al., 2019). Taken together, *L. helveticus*, *L. paracasei* and *B. infantis* upregulate the central 5-HT-BDNF system as their mutual antidepressive mechanism, although via different pathways (Figure 2B).

### Clostridium butyricum

Treatment of *C. butyricum* improved depressive-like behaviors in chronic-stressed mice. These treated mice also showed upregulated central 5-HT, BDNF and GLP-1 receptors in the brain (Sun et al., 2018). Remarkably, the combination of *C. butyricum* with antidepressants reduced depression in about 70% of treatment-resistant MDD patients, of which 30% achieved remission (Miyaoka et al., 2018). These studies support the antidepressive efficacy of non-pathogenic *C. butyricum*. It should be noted that certain strains of *C. butyricum* are pathogenic which may cause botulism and necrotizing enterocolitis (Cassir et al., 2016).

#### Butyrate, Central 5-HT-BDNF System, and GLP-1

*Clostridium butyricum*, as a resident of healthy gut microbiota, produces butyrate from carbohydrate fermentation (Araki et al., 2002; He et al., 2005; Liu J. et al., 2015). Treatment of *C. butyricum* increased central 5-HT levels and BDNF expression in mice with reduced depression (Sun et al., 2018). Another study also reported that *C. butyricum* intake upregulated neurogenesis-related pathways, such as BDNF, via butyrate production in mice (Liu J. et al., 2015). Additionally, intragastric inoculation of *C. butyricum* increased intestinal secretion of GLP-1 and the central expression of GLP-1 receptors in mice alleviated from depression (Sun et al., 2018). This effect may also be mediated by butyrate as SCFAs can bind to receptors expressed on intestinal L cells to stimulate GLP-1 secretion into the bloodstream (Tolhurst et al., 2012). GLP-1 is known for appetite and glucose control, but the activation of central GLP-1 receptors has been shown to regulate the central 5-HT system and reduce anxiety- and depressive-like behaviors in rats (Anderberg et al., 2016). Therefore, antidepressive mechanism of *C. butyricum* potentially involves a butyrate-mediated upregulation of central BDNF-5-HT system (similar to *L. paracasei* and *B. infantis*) and GLP-1 receptor expression (Figure 2B).

### Lactobacillus kefiranofaciens

*Lactobacillus kefiranofaciens* is isolated from kefir, a type of fermented milk. Oral gavage of *L. kefiranofaciens* improved behaviors of chronic-stressed, depressed mice. These treated mice also showed several physiological alterations. Levels of circulating TRP, splenic IL-10 and beneficial gut bacteria (e.g., Lachnospiraceae, Bifidobacteriaceae, and Akkermansia) increased, and KYN/TRP ratio, splenic IL-6 and IFN-γ levels and Proteobacteria abundance decreased (Sun et al., 2019). What factors mediate such broad effects of *L. kefiranofaciens* on the TRP/KYN pathway, immune system, HPA axis and gut microbiota remain unclear, but exopolysaccharide is potentially a candidate (Figure 2D).

#### Exopolysaccharide, Peripheral Inflammation, and Gut Microbiota

The only known metabolite of *L. kefiranofaciens* is an exopolysaccharide called kefiran (Maeda et al., 2004; Xing et al., 2017). The intake of kefiran modulated the gut mucosal immune system of mice (Vinderola et al., 2006), which could potentially account for changes in splenic cytokines seen in depressed mice (Sun et al., 2019). Kefiran was also shown to protect human enterocyte cell lines from adhesion and damage inflicted by toxins of pathogenic bacteria (Santos et al., 2003; Medrano et al., 2008). A further study discovered that *L. kefiranofaciens* produces a novel exopolysaccharide (not kefiran) that is bactericidal toward enteropathogens *Listeria monocytogenes* and *Salmonella enteritidis* (Jeong et al., 2017a). It may be possible that the antibacterial effects of this exopolysaccharide extend to other species in the gut microbiota. This supports the finding that *L. kefiranofaciens* supplementation ameliorated depressive-like behaviors in chronic-stressed mice by regulating gut microbiota content, which included the decreased abundance of Proteobacteria, a phylum that includes pathogens such as *Salmonella* (Sun et al., 2019). Other mice studies also supported the role of *L. kefiranofaciens* in modulating gut microbiota composition (Jeong et al., 2017b; Xing et al., 2018). Collectively, these changes in gut microbiota profile prevent gut dysbiosis that could lead to chronic inflammation, HPA axis overactivity and depression (Jeong et al., 2017b).

### Bifidobacterium breve

*Bifidobacterium breve* treatment improved symptoms of depression in innately anxious mice (Savignac et al., 2014), chronic-stressed mice (Tian et al., 2019) and schizophrenic patients with depression (Okubo et al., 2019). *B. breve* supplementation also improved mood and cognition in elderly people with mild cognitive impairment (Kobayashi et al., 2019). However, none of the accompanying physiological changes among these studies overlapped, making it difficult to identify an exact mechanism of *B. breve*. In spite of this, one study demonstrated that antidepressive mechanism of *B. breve* involves the generation of benzoic acid (Zhao et al., 2018; Figure 2D).

#### Benzoic Acid and Central Glu System

Among the 18 bacterial strains isolated from gut microbiota, *B. breve* was the most efficient converter of albiflorin to benzoic acid via microbial carboxylesterase, at the rate of 75% as compared to *L. casei*, *Lactobacillus acidophilus* and *B. longum* at about 5%. The same study further showed that orally administered benzoic acid alleviated depression in mice (Zhao et al., 2018). Benzoic acid readily crosses the intestinal barrier and BBB to inhibit D-amino acid oxidase that catabolizes D-serine, a co-agonist of *N*-methyl-D-aspartate receptor (NMDAR, a type of Glu receptor) (Zhao et al., 2018). Both D-serine and NMDARs are therapeutic targets in neuropsychiatric disorders, such as depression, schizophrenia and cognitive impairment (Durrant and Heresco-Levy, 2014). Indeed, a dysfunctional Glu system is linked to the pathophysiology of depression (Pytka et al., 2016). In line with this, *B. breve* intake increased Glu synapses in chronic-stressed mice while treating its depressive-like behaviors (Tian et al., 2019).

### Bifidobacterium longum

*Bifidobacterium longum* treatment decreased depressive-like symptoms in innately anxious mice (Savignac et al., 2014) and IBS patients with mild to moderate depression and/or anxiety (Pinto-Sanchez et al., 2017). *B. longum* supplementation also presented anxiolytic efficacy in numerous human and animal studies (Bercik et al., 2010, 2011; Allen et al., 2016; Orikasa et al., 2016). However, *B. longum* did not affect the gut inflammatory state in animals and humans, indicating a lack of immunomodulatory function (Bercik et al., 2010, 2011; Pinto-Sanchez et al., 2017). Other physiological changes, such as BDNF expression and plasma KYN/TRP ratio, seen in *B. longum*-treated mice and humans were inconsistent (Bercik et al., 2010, 2011; Orikasa et al., 2016; Pinto-Sanchez et al., 2017). Collectively, these data suggest that brain neural activity and HPA axis are possible targets of *B. longum* signaling mechanisms (Figure 2C).

#### Serpin, Central Neural Activity, and HPA Axis

Both *in vitro* and *in vivo* studies showed that *B. longum* weakened the excitability of murine myenteric neurons (Bercik et al., 2011; Khoshdel et al., 2013). Mice with inflamed intestines that were fed with *B. longum* demonstrated reduced anxiety-like behaviors, and this effect ceased upon vagotomy (Bercik et al., 2011). Intriguingly, *B. longum* intake also alleviated anxiety in colon-inflamed mice that were vagotomized before treatment (Bercik et al., 2010). The author postulated that vagus afferents are an essential conduit when *B. longum* signals enterocytes, but not colonocytes (Bercik et al., 2011). The genome of *B. longum* encodes serpin, a serine protease inhibitor (Ivanov et al., 2006; Mkaouar et al., 2016). Serpin can inhibit the activation of enteric neurons by suppressing the secretion of elastase-like proteases from IECs (Ivanov et al., 2006; Buhner et al., 2018). These studies support the premise that *B. longum* interacts with the host via the neural pathway (similar to *L. rhamnosus*). Following this, the neural activity and HPA axis of the brain may be altered. Individuals consuming *B. longum* had increased neural activity in the PFC and decreased neural activity in the amygdala and fronto-limbic regions (Allen et al., 2016; Pinto-Sanchez et al., 2017). Anomalies in the anatomy and activity of the amygdala and PFC are also commonly observed among depressed patients (Liu W. et al., 2017). Furthermore, *B. longum* intake exerted simultaneous glucocorticoids-lowering and anxiolytic effects in humans and mice (Allen et al., 2016; Orikasa et al., 2016), suggesting that *B. longum* potentially modulates the HPA axis.

### Lactobacillus gasseri

Supplementation of *L. gasseri* improved mood (Sashihara et al., 2013) and depressive-like symptoms (Sawada et al., 2017) in stressed individuals. However, no studies have evaluated the effect of *L. gasseri* on clinically depressed individuals. Interestingly, *L. gasseri* is the only dietary probiotic which showed consistent sleep-enhancing effects in humans (Nishida et al., 2017a, b; Sawada et al., 2017). Irregular sleeping patterns are frequently associated with MDD (American Psychiatric Association, 2013; Wallace and Milev, 2017), supporting the use of *L. gasseri* as a potential treatment for MDD-related sleep disturbances.

#### Gassericins, Gut Microbiota, and Parasympathetic Activity in Sleep

Stressed individuals that were given probiotic-based milk containing either heat-killed or live *L. paracasei* showed alterations in the gut microbiota profile. Heat-killed *L. gasseri* decreased *Bacteroides vulgatus* and increased *Dorea longicatena* populations (Nishida et al., 2017a), whereas live *L. gasseri* decreased growth of inflammatory Enterobacteriaceae and *Veillonella* (Sawada et al., 2017). Both studies also showed that *L. gasseri* enhanced sleep quality of participants. Another study reported that heat-killed *L. gasseri* (in milk) increased the population of *Clostridium* cluster IV group and SCFAs levels in individuals with altered bowel movements (Sawada et al., 2016). Using a similar methodology, decreased *Clostridium* cluster IV and increased *Bifidobacterium* populations were found in another group of participants (Sugawara et al., 2016). Taken together, these results suggest that heat-killed *L. gasseri* does not have a specific microbial target, but rather modifies the preexisting gut microbiota that is unique to each individual. Nevertheless, these changes in the gut microbiota composition favor an anti-inflammatory state (Sawada et al., 2016; Sugawara et al., 2016; Nishida et al., 2017a). *L. gasseri* likely alters the gut microbiota profile through its unique, heat-resistant gassericins A and T with potent antibacterial properties against enteric pathogens (Pandey et al., 2013).

Heat-killed *L. gasseri* decreased expression of leukocytic stress-responsive microRNAs and salivary cortisol levels in stressed individuals (Nishida et al., 2017b). *L. gasseri* intake also prevented downregulation of EIF2-related genes in IBS patients (Nobutani et al., 2017). These studies suggest that *L. gasseri* confers protection against detrimental effects of stress. Moreover, heat-killed *L. gasseri* intake promoted parasympathetic nerve activity while improving sleep quality of stressed individuals (Nishida et al., 2017b). In healthy individuals, administration of either live or heat-killed *L. gasseri* increased their parasympathetic activity (Otomi et al., 2015; Sugawara et al., 2016). Therefore, *L. gasseri* may modify the gut microbiota profile in such a way that lowers gut inflammation and stress response, which may consequently promote parasympathetic activity to facilitate sleep (Figure 2C).

## Challenges and Perspectives for Probiotics as Treatment for Depression

The existence of different gut microbiota compositions, depression subtypes and probiotic formulations complicate treatment outcomes and necessitate an individualized approach when using probiotics to treat depression. Despite these challenges, probiotics confer some benefits over antidepressant drugs, and there are more promising candidate probiotics that can potentially treat depression.

### Heterogeneity of Gut Microbiota Composition

Several factors are known to influence the gut microbiota composition, such as diet, medications, genetics, age, geographical location and smoking (Thursby and Juge, 2017). Recently, approximately 1000 gut-derived putative bacterial species that do not belong to any existing genus were discovered in humans (Almeida et al., 2019). Such tremendous diversity complicates the understanding of how introduced probiotics affect the overall gut microbiota. One study showed that tolerability of individuals’ gut microbiota toward the colonization of probiotics ranges from permissive to resistant (Zmora et al., 2018). This appears to depend on the baseline abundance of probiotic species in the host gut microbiota. For instance, those who were permissive toward the colonization of *Lactobacillus* had prior low levels of *Lactobacillus* populations before treatment (Zmora et al., 2018). Similarly, *B. longum* colonized the gut for a longer period in 30% of users who initially had low levels of *B. longum* (Maldonado-Gomez et al., 2016). Another study showed that the antidepressive effect of multi-species probiotics (MSP) only manifests when the administered MSP successfully colonized the gut of rats (Abildgaard et al., 2019). This is consistent with the observation that lower levels of two main probiotic genera, *Lactobacillus* and *Bifidobacterium*, are commonly found in individuals with MDD (Aizawa et al., 2016).

Despite most studies supported the effectiveness of probiotic supplements in reducing depression, not all randomized controlled trials reported the same outcome (Table 2). For instance, *L. rhamnosus* did not affect scores of anxiety, depressions, sleep, cognition, inflammatory and stress responses among healthy adults (Kelly et al., 2017). *L. rhamnosus* also did not affect perceptions of wellbeing, anxiety and stress among healthy older adults (Ostlund-Lagerstrom et al., 2016). In healthy individuals, *L. helveticus* exhibited no antidepressive effect (Chung et al., 2014; Ohsawa et al., 2018). These results imply that probiotics are less efficacious among the healthy population, which agree with a meta-analysis that reported an insignificant effect of probiotics on mood, particularly in healthy individuals (Ng et al., 2018). Therefore, probiotics could be generally more effective in colonizing gut microbiota of depressed individuals that are different from healthy people (Jiang et al., 2015; Zheng et al., 2016). In some cases, probiotic colonization may be optional for their effects to manifest. For instance, heat-killed *L. paracasei* benefited the human and animal host, in terms of neurochemical and behavioral changes (Corpuz et al., 2018; Murata et al., 2018; Wei et al., 2019). Some probiotics, such as *L. reuteri*, *L. paracasei*, *L. plantarum*, *L. gasseri*, *L. kefiranofaciens*, *B. breve*, and *B. infantis*, promoted the colonization of other beneficial microbes that contributed to the reduction of depressive-like symptoms in animals (Marin et al., 2017; Dhaliwal et al., 2018; Jang et al., 2019; Sun et al., 2019; Tian et al., 2019; Wei et al., 2019).

**TABLE 2:** Selected preclinical and clinical studies on the behavioral and physiological effects of single-species probiotics.

**Probiotic species**	**Model**	**Behavioral changes**	**Physiological changes**	**References**
*Lactobacillus rhamnosus*	Normal, healthy BALB/c male mice	↓ Anxiety ↓ Depression ↑ Memory No effect on locomotion	↓ Stress-induced ↓ in plasma CORT levels ↓ GABA_Aa2_ mRNA expression in the PFC and amygdala ↓ GABA_B1b_ mRNA expression in the HPC, amygdala and locus coeruleus ↑ GABA_Aa2_ mRNA expression in the HPC ↑ GABA_B1b_ mRNA expression in cortical regions (cingulate and prelimbic)	Bravo et al. (2011)
	BALB/c male mice subjected to MS	↓ Depression	↓ Stress-induced ↑ in plasma CORT levels ↑ Recovery toward basal corticosterone levels	McVey Neufeld et al. (2018)
	Healthy human males (aged 22–33, mean ≈ 23–25 years)	No effect on mood and anxiety	No changes in cortisol response to stress, plasma levels of IL10, IL1β, IL6, IL8 and TNFα, and whole blood levels of TLR-4	Kelly et al. (2017)
	Pregnant women (14–16 weeks gestation)	↓ Anxiety ↓ Depression	N/A	Slykerman et al. (2017)
	(With prebiotics) Obese individuals (aged 18–55, mean ≈ 35–58 years)	↓ Depression ↓ Food cravings ↑ Satiety	N/A	Sanchez et al. (2017)
*Lactobacillus casei* strain Shirota	Healthy middle-age human adults (aged 48–79, mean ≈ 62 years)	↓ Depression in those with low mood	N/A	Benton et al. (2007)
	Individuals with chronic fatigue syndrome (aged 18–65 years)	↓ Anxiety No effect on depression	↑ Fecal *Lactobacillus* and *Bifidobacteria* populations	Rao et al. (2009)
	Healthy students under stressful examination (aged < 40, mean ≈ 23 years)	↓ Stressful feelings No effect on anxiety	↓ Salivary cortisol levels ↓ Gastrointestinal symptoms ↓ Fecal *Bacteroidaceae* populations ↑ Diversity of the gut microbiota Prevented changes in expression of approx. 100 stress-responsive genes	Kato-Kataoka et al. (2016)
*Lactobacillus brevis*	Sprague–Dawley male depressed rats	↓ Depression	N/A	Ko et al. (2013)
	ICR male mice	↑ Sleep duration	N/A	Han et al. (2017)
	C3H-HeN male mice	↑ Sleep duration ↑ Wheel-running	N/A	Miyazaki et al. (2014)
*Lactobacillus reuteri*	C57BL/6J, C57BL/6N, and BALB/cJ male mice subjected to CUMS	↓ Depression	↓ Stress-induced ↑ in intestinal IDO1 expression ↓ Stress-induced ↑ in KYN levels ↑ Stress-induced ↓ in fecal H_2_O_2_ levels ↑ Stress-induced ↓ in *Lactobacillus* populations	Marin et al. (2017)
	C57BL/6 male mice subjected to immobilization stress	↓ Anxiety ↓ Depression	↓ Stress-induced ↑ in activated microglia infiltration into the HPC ↓ Stress-induced ↑ in colon shortening, myeloperoxidase activity and IL-6 expression in the colon ↓ Stress-induced ↑ in blood CORT, IL-6, and LPS levels ↓ Stress-induced colitis ↓ Stress-induced ↑ in Proteobacteria populations ↑ Stress-induced ↓ in HPC BDNF expression ↑ Stress-induced ↓ in Bacteroidetes, Firmicutes, and Actinobacteria populations	Jang et al. (2019)
*Lactobacillus plantarum*	MS vs. naïve male C57BL/6J mice	↑ Locomotion In naïve mice: ↓ Anxiety In MS mice: ↓ Depression ↓ Anhedonia	↓ Stress-induced ↑ in CORT in MS mice ↑ DA, DOPAC, and HVA in the PFC of MS and naïve mice ↓ 5-HIAA and no change in 5-HT levels in the PFC of MS mice ↑ 5-HT levels in the PFC of naïve mice ↓ 5-HIAA levels in the PFC of naïve mice ↑ IL-10, ↓ IL-6 and no effect on TNF-α levels in the serum of MS mice	Liu Y.W. et al. (2016)
	Germ-free C57BL/6JN male mice	↓ Anxiety ↑ Locomotion No effect on depression	↑ 5-HT and DA levels in the striatum, but not the PFC or HPC No effects on serum GR levels	Liu W.H. et al. (2016)
	Swiss albino male mice subjected to CUMS or sleep-deprivation stress	↓ Anxiety ↓ Depression ↑ Memory ↑ Learning ↑ Locomotion	↓ Stress-induced ↑ in malonaldehyde, MAOs and nitrate levels in the brain ↓ Stress-induced ↑ in serum levels of TNF-α, CORT, and LPS ↑ Stress-induced ↓ in glutathione and HPC BDNF levels ↑ Abundance of *Lactobacillus* ↓ Stress-induced ↓ abundance of *Bacteroidetes* and *Roseburia* ↑ Fecal acetic and butyric acid levels Prevented stress-induced ↑ in permeability of BBB and intestinal barrier, and *Enterobacteriaceae* levels	Dhaliwal et al. (2018)
	MDD patients undergoing SSRI medications (mean age ≈ 39 years)	↑ Memory ↑ Attention ↑ Learning No effect on depression and stress	↓ Plasma KYN levels ↑ 3-hydroxykynurenine/KYN ratio No changes in plasma levels of TNF-α, IL-6, IL-1β, and cortisol	Rudzki et al. (2019)
	Stressed human adults with mild levels of depression (aged 18–60, mean ≈ 31 years)	↓ Anxiety ↓ Stress ↑ Memory ↑ Learning ↓ Depression (not stat. sig)	↓ Plasma IFN-γ and TNF-α levels ↓ Plasma IL-1β and cortisol levels (not stat. sig.)	Lew et al. (2018)
*Faecalibacterium prausnitzii*	Sprague–Dawley male rats subjected to CUMS	↓ Anxiety ↓ Depression	↓ Stress-induced ↓ in plasma levels of CORT, CRP, and IL-6 ↑ SCFAs levels in the cecum ↑ Stress-induced ↓ in plasma IL-10 levels	Hao et al. (2019)
*Lactobacillus helveticus*	Sprague–Dawley male rats subjected to chronic restraint stress	↓ Anhedonia ↓ Anxiety ↑ Locomotion ↑ Memory	↓ Stress-induced ↑ in CORT and adrenocorticotropic hormone levels ↓ Stress-induced ↓ in plasma IL-10 levels ↑ Stress-induced ↓ in HPC BDNF expression ↑ Stress-induced ↓ in 5-HT and NE levels in the HPC No changes in stress-induced ↓ in plasma IFN-γ and TNF-α levels	Liang et al. (2015)
	Sprague–Dawley male rats with hyperammonemia-induced neuroinflammation	↓ Anxiety ↑ Memory ↑ Learning	↓ Stress-induced ↑ in KA/KYN ratio ↓ Stress-induced ↑ in PGE2 levels in the cerebellum and HPC ↓ Stress-induced ↑ in IL-1β levels in the cerebellum, HPC, and PFC ↓ 5-HT levels in the cerebellum and HPC ↑ Stress-induced ↓ in KYN/TRP ratio No effect in stress-induced ↑ in 5-HIAA levels in the HPC, cerebellum, and PFC	Luo et al. (2014)
	C57BL/6J male mice subjected to sub-chronic social defeat stress	↓ Anhedonia ↓ Anxiety	↑ Stress-induced ↓ in dopamine D3 and serotonin 1A receptors expression Restore stress-induced changes in gene expression in the nucleus accumbens No effects on serum CORT levels and gut microbiota composition	Maehata et al. (2019)
	Healthy elderly humans (aged 60–75, mean ≈ 65 years)	↑ Memory ↑ Attention ↑ Learning No effects on stress levels and depression	No effects on plasma levels of BDNF and whole blood viscosity	Chung et al. (2014)
	Healthy middle-aged humans (aged 50–70, mean ≈ 58 years)	↑ Memory ↑ Attention No effects on depression	N/A	Ohsawa et al. (2018)
*Lactobacillus paracasei*	CORT-induced depressed male C57BL/6J mice (live or heat-killed *L. paracasei)*	↓ Depression ↓ Anhedonia ↓ Anxiety	↑ Stress-induced ↓ abundance of *Bifidobacterium* (live) ↑ Stress-induced ↓ in 5-HT levels in the HPC, PFC, and striatum (live) ↑ Stress-induced ↓ DA levels in the HPC and PFC (heat-killed) ↑ Stress-induced ↓ in BDNF levels and MR and GR receptors expression in the HPC No effect on serum CORT levels, both basal and in response to stress	Wei et al. (2019)
	Senescence-accelerated female SAMP8 mice (heat-killed *L. paracasei*)	Prevented age-related cognitive decline	↓ 5-HT-degrading enzymes, particularly MAOA, levels in the HPC ↑ 5-HT levels in brain tissues and serum ↓ BDNF expression and CREB phosphorylation in the HPC No effect on the gene expression of 5-HT-synthesis-related enzyme	Corpuz et al. (2018)
	Senescence-accelerated male and female SAMP8 mice (live *L. paracasei*)	Prevented age-related cognitive decline and anxiety	↓ Serum TNF-α and MCP-1 levels ↑ Levels of DA, DC, 5-HT and 5-HIAA levels in the striatum and HPC ↑ Levels of serum BDNF, IL-10, SOD, and GPx	Huang et al. (2018)
	Healthy females under examination stress (heat-killed *L. paracasei*) (aged > 18, mean ≈ 21 years)	Prevented decline in mood and immunity	↓ Frequency of common cold in those susceptible No effect on salivary secretory IgA concentrations	Murata et al. (2018)
*Bifidobacterium infantis*	Naïve Sprague–Dawley male rats	No effect on depression	↓ Plasma IFN-γ, TNF-α, IL-10, and IL-6 levels ↓ 5-HIAA levels in the frontal cortex ↓ DOPAC levels in the amygdaloid cortex ↓ NE levels in the HPC (not stat. sig.) ↑ Plasma TRP and KYN levels No effects in baseline CORT levels	Desbonnet et al. (2008)
	Sprague Dawley male rats subjected to MS	↓ Depression	↓ Stress-induced ↑ in plasma IL-6 and corticotrophin-releasing factor mRNA expression in the amygdala ↑ Stress-induced ↓ in NE levels in the brainstem No effects on plasma TRP/KYN ratio and baseline CORT concentrations	Desbonnet et al. (2010)
	Male adult C57BL/6J mice subjected to CUMS	↓ Depression ↓ Anhedonia ↓ Anxiety	↓ Stress-induced ↑ Veillonellaceae and *Desulfovibrio* populations ↑ 5-HT and 5-HTP levels in the HPC ↑ Expression of Tph1 mRNA in RIN14B cells (*in vitro*) ↑ BDNF levels in the PFC ↑ Stress-induced ↓ in cecum butyrate levels ↑ Alpha diversity of gut microbiota ↑ Glutamatergic synapse ↑ Phenylalanine/tyrosine/TRP biosynthesis No effect on spleen regulatory T cells	Tian et al. (2019)
*Clostridium butyricum*	C57BL/6 male mice subjected to CUMS	↓ Depression No effect on locomotion	↑ Stress-induced ↓ in brain levels of 5-HT and BDNF ↑ Stress-induced ↓ in intestinal GLP-1 secretion and cerebral expression of GLP-1 receptor	Sun et al. (2018)
	(With SSRIs or SNRIs) Treatment-resistant MDD patients (mean age ≈ 42–44 years)	↓ Depression	N/A	Miyaoka et al. (2018)
*Lactobacillus kefiranofaciens*	Kunming male mice subjected to CUMS	↓ Depression ↓ Anhedonia	↓ Stress-induced ↑ in serum CORT levels and KYN/TRP ratio ↓ IL-6 and IFN-γ levels in the spleen ↓ Abundance of Proteobacteria ↑ Stress-induced ↓ serum TRP levels ↑ IL-10 levels in the spleen ↑ Abundance of anti-inflammatory Actinobacteria, Bacteroidetes, Lachnospiraceae, Coriobacteriaceae, Bifidobacteriaceae, and Akkermansia	Sun et al. (2019)
*Bifidobacterium breve*	Innately anxious BALB/c male mice	↓ Depression ↓ Anxiety No effect on locomotion	No effect on CORT levels, both baseline and in response to stress	Savignac et al. (2014)
	Male adult C57BL/6J mice subjected to CUMS	↓ Depression ↓ Anhedonia ↓ Anxiety	↓ Chronic stress-induced CORT release ↓ Stress-induced ↑ Veillonellaceae populations ↑ Expression of Tph1 mRNA in RIN14B cells (*in vitro*) ↑ BDNF levels in the PFC ↑ Stress-induced ↓ in alpha diversity of the gut microbiota ↑ Glutamatergic synapse ↑ Phenylalanine/tyrosine/TRP biosynthesis No effect on spleen regulatory T cells	Tian et al. (2019)
	Schizophrenic individuals with anxiety and depression (aged > 20, mean ≈ 41–46)	↓ Depression ↓ Anxiety	↑ Relative abundance of Parabacteroides ↑ Serum IL-22 and TRANCE expression No effects on *Bifidobacterium* populations and serum levels of IL-6 and TNF-α	Okubo et al. (2019)
	Elderly humans with mild cognitive impairment (mean age ≈ 83 years)	↑ Mood ↑ Memory ↑ Attention ↑ Learning	N/A	Kobayashi et al. (2019)
*Bifidobacterium longum*	Innately anxious BALB/c male mice	↓ Depression ↓ Anxiety No effect on locomotion	No effect on CORT levels, both baseline and in response to stress	Savignac et al. (2014)
	Healthy human males (aged 18–40, mean ≈ 25 years).	↓ Stress ↓ Anxiety ↓ Memory ↓ Attention ↑ Learning	↓ Salivary cortisol output and anxiety scores in response to stressor ↑ Neural activity of the PFC	Allen et al. (2016)
	IBS patients with mild to moderate depression and/or anxiety (median age = 40 and 46.5 years)	↓ Depression ↑ Life quality No effect on anxiety	↓ Responses to negative emotional stimuli in the amygdala and fronto–limbic regions ↓ Urine levels of methylamines and aromatic amino acids metabolites No effect on fecal microbiota profiles, serum inflammatory markers (CRP, TNF-α, IFN-γ, IL-1β, IL-6, IL-8, IL-10, IL12), BDNF, substance P and 5-HT levels	Pinto-Sanchez et al. (2017)
*Lactobacillus gasseri*	University male students with daily strenuous exercise (aged < 30, mean ≈ 20 years)	↑ Mood in depressed individuals	Prevent stress-induced ↓ in natural killer cell activity	Sashihara et al. (2013)
	Medical (cadaver dissection course) male students (aged 24)	↓ Depression ↓ Anxiety ↑ Sleep quality	↓ Salivary cortisol release ↓ Growth of inflammatory Enterobacteriaceae and *Veillonella* Prevented the downregulation of EIF2-related genes of peripheral leukocytes	Sawada et al. (2017)
	Medical (cadaver dissection course) students (heat killed *L. gasseri*) (aged 18–34, mean ≈ 21 years)	In men: ↓ Sleep latency ↑ Sleep duration In women: ↓ Somatic symptoms	↓ Diarrhoea-like symptoms (in men) ↑ Fecal *Bacteroides vulgatus* levels ↓ Fecal *Dorea longicatena* levels No effect on salivary stress markers (cortisol, CgA, and alpha amylase levels)	Nishida et al. (2017a)
	Medical students in pre-examination (heat-killed *L. gasseri*) (mean age ≈ 25 years)	↓ Sleep latency ↓ Sleep awakenings	↑ Ratio of parasympathetic/sympathetic nerve activity ↑ Stage N3 in the non-REM sleep period ↓ Stress-induced ↑ in salivary cortisol levels ↓ Stress-induced ↑ expression of stress-responsive microRNAs	Nishida et al. (2017b)

### Heterogeneity of Depression

Major depressive disorder is characterized by depressed mood and/or anhedonia, in addition to excessive guilt, suicidal ideation, changes in appetite and sleep, psychomotor retardation, poor concentration and fatigue (American Psychiatric Association, 2013). From these diagnostic criteria, approximately a thousand combinations of symptoms (Ostergaard et al., 2011) and 19 depression subtypes (Harald and Gordon, 2012; Sharpley and Bitsika, 2014) can be derived. These subtypes of depression are often grouped as a single term, namely depression, which should not be the case when evaluating therapeutic potential of probiotics.

Some associations can be drawn by matching behavioral benefits of probiotics to the characteristics of depression subtypes (Table 2). For instance, the sucrose preference test in rodents reflects the anhedonia subtype (Dedic et al., 2011). Probiotics that have been shown to improve the outcome of this test include *L. helveticus* (Liang et al., 2015), *L. plantarum* (Liu Y.W. et al., 2016), *L. paracasei* (Wei et al., 2019), *L. kefiranofaciens* (Sun et al., 2019), *B. infantis* (Tian et al., 2019), and *B. breve* (Tian et al., 2019). Among these probiotics, *L. plantarum* (Liu Y.W. et al., 2016) and *L. paracasei* (Wei et al., 2019) also modulated the central DA system, whereas *B. infantis* and *B. breve* upregulated tyrosine (precursor to DA) biosynthesis (Tian et al., 2019). An impaired DA system represents the hallmark pathophysiology of anhedonia (Dunlop and Nemeroff, 2007). This provides a proof of concept that these probiotics may be effective in treating anhedonia.

Somatic depression subtype is characterized by psychomotor agitation/retardation (i.e., locomotion), changes in weight/appetite, insomnia/hypersomnia and fatigue without physical exertion (Sharpley and Bitsika, 2014). Probiotics that improved locomotor activity of rodents include *L. plantarum* (Liu W.H. et al., 2016; Dhaliwal et al., 2018), *L. helveticus* (Liang et al., 2015) and *L. brevis* (Miyazaki et al., 2014). Intake of *L. brevis* increased sleep duration in healthy mice (Miyazaki et al., 2014; Han et al., 2017), and *L. gasseri* enhanced sleep quality in medical students with mild depression (Nishida et al., 2017a, b). *L. rhamnosus* supplementation modulated appetite-associated genes and attenuated appetite in zebrafish (Falcinelli et al., 2016, 2017). In combination with prebiotics, *L*. *rhamnosus* exerted antidepressive effect and appetite control in obese individuals (Sanchez et al., 2017). Hence, symptoms of somatic depression are rather distinct and may be improved differently with different probiotics.

Cognitive depression subtype is distinguished by poor concentration and memory function as well as indecisiveness (Sharpley and Bitsika, 2014). Behavioral assessments for memory function in mice include the Morris water maze, Barnes maze and other behavioral tests (Dedic et al., 2011). Administration of probiotics including *L. helveticus* (Ohland et al., 2013; Luo et al., 2014; Liang et al., 2015), *L. plantarum* (Dhaliwal et al., 2018), and *L. paracasei* (Corpuz et al., 2018; Huang et al., 2018) enabled animals to perform these memory test more effectively. Attention, memory and learning behaviors in humans are assessed by cognitive tests, such as the Stroop, verbal-learning and digit-symbol tests. Improvements in these tests have been shown with the intake of (1) *L. helveticus* (Chung et al., 2014; Ohsawa et al., 2018) and *B. longum* (Allen et al., 2016) in healthy adults; (2) *L. plantarum* in MDD patients (Rudzki et al., 2019) and stressed adults with mild depression (Lew et al., 2018); and (3) *B. breve* in elderly with mild cognitive impairment (Kobayashi et al., 2019). Thus, some probiotics appear to improve cognition regardless of depression.

Anxious depression subtype refers to major depression that comorbid with high levels of anxiety (Harald and Gordon, 2012). In mice, anxiety can be measured by behavioral tests, such as the elevated plus maze and open field tests (Dedic et al., 2011). In humans, anxiety is generally assessed with questionnaires. Probiotics that exhibit anxiolytic effect include *L. rhamnosus* (Bravo et al., 2011; Bharwani et al., 2017; McVey Neufeld et al., 2017; Slykerman et al., 2017), *L. helveticus* (Ohland et al., 2013; Luo et al., 2014; Liang et al., 2015), *L. plantarum* (Liu W.H. et al., 2016; Liu Y.W. et al., 2016; Dhaliwal et al., 2018; Lew et al., 2018), *B. longum* (Bercik et al., 2010, 2011; Savignac et al., 2014; Allen et al., 2016) and *B. breve* (Savignac et al., 2014; Okubo et al., 2019; Tian et al., 2019). Moreover, MSPs intake often decreased depression and anxiety simultaneously in randomized controlled trials (Mohammadi et al., 2016; Kouchaki et al., 2017; Jamilian et al., 2018; Raygan et al., 2018; Ostadmohammadi et al., 2019; Salami et al., 2019).

Conventional SSRIs that target the 5-HT system often fail to treat anhedonic patients and, in some cases, worsen their symptoms (Dunlop and Nemeroff, 2007). Antidepressant drugs (e.g., SSRI and SNRI) are also ineffective against other depression subtypes, namely the somatic (Tylee and Gandhi, 2005), cognitive (Shilyansky et al., 2016) and anxious depression (Ionescu et al., 2014). Therefore, certain probiotics may serve as an adjuvant or alternative treatment for MDD and its subtypes. A pilot study showed that MSP, together with a magnesium supplement, decreased depression in SSRI treatment-resistant patients (Bambling et al., 2017). A clinical trial also reported that the combination of *B. longum* and *L. helveticus* decreased depression in MDD patients with prior use of standard antidepressants (Kazemi et al., 2019).

### Single-Species and Multi-Species Probiotic

In studies that investigated behavioral effects of probiotics, about 60% of animal studies and 50% of human studies used single-species probiotics (SSPs) (Joseph and Law, 2019). Studies with SSPs promote a better understanding of the function and contribution of individual probiotic, which is difficult to measure in MSPs. However, MSPs may have higher potency in humans. In MDD patients, SSP (*L. plantarum*) did not reduce depression but improved cognition (Lew et al., 2018), whereas MSPs had repeatedly shown antidepressive efficacy (Akkasheh et al., 2016; Bambling et al., 2017; Ghorbani et al., 2018; Kazemi et al., 2019). MSPs often gave better therapeutic efficacy compared to that of SSPs in gut-related disorders and pathogen infections, which could be explained by an overall higher dosage (Chapman et al., 2011, 2012). Indeed, MSPs with a higher dosage improved symptoms of depression and anxiety in healthy individuals compared to that of a lower dosage (Tran et al., 2019). MSPs are also hypothesized to exhibit synergistic effects that would have an expanded effect on the host physiology (Chapman et al., 2012). In contrast, SSPs are speculated to promote better colonization as it does not have to compete for nutrient or adhesion sites in the host (Chapman et al., 2011). This highlights the need for more studies to understand how probiotics in MSPs interact with each other and with existing gut microbiota, and which probiotic(s) is suitable in formulation of MSPs for antidepressive efficacy.

### Advantages of Probiotics as Antidepressive Treatment

Probiotics are generally safe for consumption, except for immune-compromised and critically sick individuals wherein probiotics may cause sepsis, pneumonia, endocarditis and allergies (Didari et al., 2014). Still, it has been viewed by some that more human trials are required to establish the dosage efficacy and long-term safety profile of probiotics (Kothari et al., 2018). For antidepressant drugs such as SSRIs, side effects occur in 40-60% of users which include sexual dysfunction, suicidality, emotional numbness and addiction (Read and Williams, 2018). A meta-analysis data showed that users of antidepressant drugs were associated with a 33% increased risk of mortality (Maslej et al., 2017). On the other hand, probiotics possess fewer side effects than antidepressant drugs. For instance, rats fed with *L. brevis*-fermented milk exhibited comparable antidepressive efficacy to fluoxetine-treated rats, but without side effects of fluoxetine (decreased appetite and weight loss) (Ko et al., 2013).

Antidepressant usage is also associated with stigma, such as being perceived as emotionally weak and dependent on drugs, which contributes to the disease severity and poor adherence to treatment (Castaldelli-Maia et al., 2011). In a survey study, 77% of depressed patients prefer to hide their use of antidepressant medication from others (Martinez et al., 2018). However, the prevalence of perceived stigma against antidepressants differs based on the population studied (Castaldelli-Maia et al., 2011). To this end, probiotics may help as an alternative treatment for depression, given that probiotics have not been associated with any perceived social stigma (Wallace and Milev, 2017).

### Candidate Probiotics With Potential Antidepressive Effect

*Bifidobacterium pseudocatenulatum* is known for its regulation of obesity-related changes in metabolism and the immune system (Cano et al., 2013; Moya-Perez et al., 2014, 2015; Sanchis-Chorda et al., 2018). *B. pseudocatenulatum* intake reversed diet-induced obesity, depression, high corticosterone and low hippocampal 5-HT levels in mice (Agusti et al., 2018). However, a high-fat diet model is meant to study the pathophysiology of obesity and type 2 diabetes (Winzell and Ahren, 2004; Wang and Liao, 2012). It is, thus, unclear if *B. pseudocatenulatum* would decrease depression in mice without obesity. Another study showed that anxiety-like behaviors diminished in chronic-stressed mice fed with *B. pseudocatenulatum*, but depressive-like behaviors were unevaluated (Moya-Perez et al., 2017). Therefore, further studies are required to determine whether *B. pseudocatenulatum* has an independent antidepressive effect.

*Bacillus coagulans* supplementation relieved symptoms of both IBS and depression in patients diagnosed with IBS and MDD. This clinical recovery is accompanied by a decrease in serum myeloperoxidase, an inflammatory marker (Majeed et al., 2018). However, patients might have experienced less depression as a result of reduced IBS symptoms. Interestingly, *B. coagulans* intake increased levels of circulating IL-10, fecal *F. prausnitzii* and SCFAs in older adults (Nyangale et al., 2014, 2015). As *F. prausnitzii* and butyrate are associated with antidepressive properties (Hao et al., 2019), *B. coagulans* may also indirectly reduce depression and improve gut health.

*Bifidobacterium bifidum* and *L. acidophilus* were often included in the formulation of MSPs to treat depressive symptoms in patients with MDD (Akkasheh et al., 2016; Bambling et al., 2017; Ghorbani et al., 2018) and other health conditions, such as polycystic ovarian syndrome, multiple sclerosis and IBS (Kouchaki et al., 2017; Ostadmohammadi et al., 2019; Zhang et al., 2019). Surprisingly, *B. bifidum* and *L. acidophilus* have not been tested independently for its antidepressive effect. *B. bifidum* intake improved mood and reduced symptoms of abdominal pain, diarrhea and constipation in patients with gastrointestinal disorders (Urita et al., 2015). However, the mood elevation could be due to recovery of gastrointestinal symptoms rather than effect of probiotics solely. Both *in vitro* and *in vivo* models showed that *L. acidophilus* protects the intestinal barrier integrity by preventing pathogen adherence and release of proinflammatory cytokines (Chen et al., 2009; Justino et al., 2015; Alamdary et al., 2018; Lepine et al., 2018; Najarian et al., 2019). Taken together, *B. bifidum* and *L. acidophilus* potentially exhibit antidepressive effect and their direct influence on depression warrants further investigation.

*Bacteroides fragilis* has been proposed as a potential probiotic, although its pathogenicity needs to be taken into consideration. *B. fragilis* secretes polysaccharide A and expresses sphingolipids that benefit the host gut health and immune system (Troy and Kasper, 2010; Tan et al., 2019). *Bacteroides* genus is likely to be the largest GABA producer amongst human gut microbiota, with *B. fragilis* produces GABA at low pH. The same study also found that neural patterns of a typical MDD patient correlated with low fecal levels of *Bacteroides* (Strandwitz et al., 2019). Hence, antidepressive potential of *B. pseudocatenulatum*, *B. coagulans*, *B. bifidum*, *L. acidophilus*, and *B. fragilis* warrants further investigation. It is also worth noting that *Bifidobacterium adolescentis*’s antidepressive capability may be a new probiotic candidate (Jang et al., 2019). Evidently, an increasing number of probiotics are being presented as a potential treatment for depression. This provides a wide repository of available probiotics, with different species combinations, that can be assessed for clinical efficacy against depression.

## Conclusion

The MGB axis enables the bidirectional communication between the gut microbiota and the brain. When this axis becomes maladaptive, the host physiology is adversely affected which may lead to the development of depression. Probiotics have shown clinical efficacy in the treatment of depression by modulating the MGB axis. Yet, the complexity of gut microbiota and heterogeneity of depression presents a challenge to explain the underlying mechanisms that contribute to this clinical efficacy. Nonetheless, cumulative evidence suggests the therapeutic potential of probiotics for certain depression subtypes, with fewer side effects and less stigma compared to standard antidepressants.

Limitations of this review include: (1) inferences of probiotic mechanisms were derived from preclinical and *in vitro* data; (2) interactions of probiotics with other members of gut microbiota were unexplored, therefore the mechanisms of MSPs was unable to be explored; (3) strain-specific effects of bacterial species were neglected; (4) potential applications for probiotics for depression subtypes are hypothesized, however, clinical evidence is limited; (5) effect sizes of probiotics as antidepressants was not evaluated. Notwithstanding these caveats, this review adds further understanding to the potential antidepressive effects and therapeutic potentials of probiotics. Venema (2017) stated that it is imperative to grasp the underlying molecular mechanisms of the MGB axis, and which microbial populations are pertinent for this intervention, to advance the marketability of probiotics.

## Author Contributions

SY wrote and edited the manuscript. TT, JC, and WL conceptualized and edited the manuscript. All authors contributed to the intellectual input and critical revision of the manuscript.

## Conflict of Interest

The authors declare that the research was conducted in the absence of any commercial or financial relationships that could be construed as a potential conflict of interest.

## Abbreviations

5-HIAA: 5-hydroxyindoleacetic acid
5-HT: 5-hydroxytryptamine (serotonin)
5-HTP: 5-hydroxytryptamine
BBB: blood-brain barrier
BDNF: brain-derived neurotropic factor
CgA: salivary chromogranin A
CORT: corticosterone
CREB: cAMP response element binding protein
CRP: C-reactive protein
CUMS: chronic unpredictable mild stress
DA: dopamine
DC: dihydroxyphenylacetic acid
EIF2: eukaryotic initiation factor 2
GABA: gamma-aminobutyric acid
GLP-1: glucagon-like peptide-1
GPx: glutathione peroxidase
GR: glucocorticoid
H_2_O_2_: hydrogen peroxide
HPC: hippocampus
HVA: homovanillic acid
IBS: irritable bowel syndrome
IDO: indolamine 2,3-dioxyhydrogenase
IFN: interferon
IgA: immunoglobin A
IL: interleukin
KA: kynurenic acid
KYN: kynurenine
LPS: lipopolysaccharides
MAOA: monoamine oxygenase A
MCP-1: monocyte chemotactic protein-1
MDD: major depressive disorder
MR: mineralocorticoid
MS: maternal separation model
NE: norepinephrine
PFC: prefrontal cortex
PGE2: prostaglandin E2
REM: rapid eye movement
SCFA: short-chain fatty acids
SNRI: serotonin-noradrenaline reuptake inhibitor
SOD: superoxide dismutase
SSRI: selective serotonin reuptake inhibitor
TLR: toll-like receptor
TNF-α: tumor necrosis factor-α
Tph1: tryptophan hydroxylase 1
TRANCE: TNF-related activation-induced cytokine
TRP: tryptophan.
